# Characterization and Functional Analysis of Five MADS-Box B Class Genes Related to Floral Organ Identification in *Tagetes erecta*

**DOI:** 10.1371/journal.pone.0169777

**Published:** 2017-01-12

**Authors:** Ye Ai, Chunling Zhang, Yalin Sun, Weining Wang, Yanhong He, Manzhu Bao

**Affiliations:** 1 Key Laboratory of Horticultural Plant Biology, Ministry of Education, College of Horticulture and Forestry Sciences, Huazhong Agricultural University, Wuhan, Hubei, China; 2 College of Landscape Architecture, Fujian Agriculture and Forestry University, Cangshan District, Fuzhou, Fujian, China; 3 Institute of Vegetable Science, Wuhan Academy of Agricultural Sciences, Wuhan, Hubei, China; 4 Gulf Coast Research and Education Center, Department of Environmental Horticulture, University of Florida, Wimauma, Florida, United States of America; University of Naples Federico II, ITALY

## Abstract

According to the floral organ development ABC model, B class genes specify petal and stamen identification. In order to study the function of B class genes in flower development of *Tagetes erecta*, five MADS-box B class genes were identified and their expression and putative functions were studied. Sequence comparisons and phylogenetic analyses indicated that there were one *PI*-like gene—*TePI*, two *euAP3*-like genes—*TeAP3*-*1* and *TeAP3*-*2*, and two *TM6*-like genes—*TeTM6*-*1* and *TeTM6*-*2* in *T*. *erecta*. Strong expression levels of these genes were detected in stamens of the disk florets, but little or no expression was detected in bracts, receptacles or vegetative organs. Yeast hybrid experiments of the B class proteins showed that TePI protein could form a homodimer and heterodimers with all the other four B class proteins TeAP3-1, TeAP3-2, TeTM6-1 and TeTM6-2. No homodimer or interaction was observed between the euAP3 and TM6 clade members. Over-expression of five B class genes of *T*. *erecta* in *Nicotiana rotundifolia* showed that only the transgenic plants of *35S*::*TePI* showed altered floral morphology compared with the non-transgenic line. This study could contribute to the understanding of the function of B class genes in flower development of *T*. *erecta*, and provide a theoretical basis for further research to change floral organ structures and create new materials for plant breeding.

## Introduction

*Tagetes erecta* is a commercial plant renowned for its significant ornamental, industrial and medicinal usage [1–3]. It is a member of the Asteraceae which is characterized by the structure of a terminal capitulum. Its unique inflorescence comprises hundreds of florets of two types, ray florets in the periphery and disk florets in the center. The ray florets have three whorls of floral organs (sepal, petal, and carpel), while the disk florets have four whorls of floral organs (sepal, petal, stamen and carpel). Complexity of the capitulum structure greatly hinders manual emasculation, a necessity in plant breeding methodology. Male sterile plants with defective anthers and degenerated petals of ray and disc florets could provide a cost-effective alternative for plant breeders [4, 5]. Advancing technology of molecular biology has the potential to modify floral structure for easier artificial breeding, yet literature related to the molecular mechanisms of floral organ development in *T*. *erecta* is lacking.

Based on the study of homeotic mutants in the model plants *Arabidopsis thaliana* and *Antirrhinum majus*, the classical ABC model was proposed to explain the genetic regulation of floral organ development, in which the identity of each floral whorl component is determined by a combination of genes grouped into three homeotic functional classes, called class A, B and C genes, respectively [6, 7]. Class A genes specify sepals of the outermost whorl; class A and B genes collectively control petal identity in the second whorl; class B and C genes concomitantly specify stamens in the third whorl; class C genes determine formation of the carpels in the fourth whorl. Subsequent discoveries of the class D and E genes expanded the model to its contemporary form, the ABCDE model [8]. Class D genes regulate ovule development [9, 10], while class E genes specify ovule identity and are necessary for specification of sepal, petal, stamen and carpel identities when functioning together with class A, B, C, and D genes, respectively [11, 12].

In view of the original characterization in *A*. *thaliana* (Brassicaceae) and *A*. *majus* (Plantaginaceae), B class genes could be divided into *APETALA 3* (*AP3*) / *DEFICIENS* (*DEF*) and *PISTILLATA* (*PI*) / *GLOBOSA* (*GLO*) subfamilies [13, 14]. Kim et al. [15] studied the phylogeny and diversification of B-function MADS-box genes in angiosperms, suggesting that a duplication event happened approximately 260 million years ago was responsible for producing the *PI/GLO* and *AP3/DEF* lineages. The estimated timeframe placed the duplication event past the splitting of extant gymnosperms and angiosperms, but well before the oldest angiosperm fossils. In the higher eudicot species, the *AP3/DEF* subfamily experienced another significant duplication event and was divided into the eu*AP3* and *Tomato MADS-box gene 6* (*TM6*) lineages [16]. These three lineages can be distinguished according to the different C-terminal motifs. The PI/GLO-like proteins have a PI-motif, while the euAP3 and TM6-like proteins have the euAP3 and paleoAP3 motifs, respectively. Both the euAP3 and TM6-like proteins have a PI-motif-derived region [16–18].

Many studies on MADS-box genes indicate the ABC model is not only applicable to the eudicot plants but also to monocot plants [19]. In both eudicots and monocots, B class genes play similar roles in specifying petal and stamen development [20–26]. Functional analysis in *Gerbera hybrida* (Asteraceae) showed that the *PI/GLO* and *euAP3* lineage genes encoded the classical B function and the *TM6* lineage gene might act redundantly in stamen development. Down-regulating the expression of the *PI/GLO* (*GGLO1)* and eu*AP3* (*GDEF2*) lineage genes led to stamen and petal defects [27]. In *Helianthus annuus* (Asteraceae), the B class genes encoded the B function and were involved in the formation of petals and stamens. Furthermore, it was found that *HaPI* and *HaAP3* were preferentially expressed in petals of ray flowers but their expression levels were significantly weaker in petals and stamens of disk flowers [28].

In this study, expression and putative functions of five *T*. *erecta* B class genes were studied. This work provides a better understanding of B class gene function in floral organ development of *T*. *erecta*, and provides a theoretical basis useful in modifying floral organ structure and generating new germplasm resources for plant breeders.

## Materials and Methods

### Materials

Inbred line TE-I-1 of *T*. *erecta* had been developed through 10 generations of self-crossing, which had one whorl of ray florets in the periphery of the capitulum. The plants were grown in 21 cm sized pots in natural conditions in fall 2012 at Huazhong Agricultural University, Wuhan, Hubei Province, China (lat. 30°28'36.5" N, long, 114°21'59.4" E).

### RNA extraction of various tissues and organs

When the plants were in florescence phase, samples of roots, tender stems, fresh leaves, different sizes of flower buds (1 mm, 3 mm, 5 mm and 7 mm in diameter, respectively), sepals, petals and pistils of ray and disk florets, stamens of disk florets, receptacles, bracts, and ovaries of opened flowers were collected, frozen immediately in liquid nitrogen and stored at -80°C for further RNA extraction. Total RNA was isolated using Trizol reagent (Tiangen, Shanghai, China) according to manufacturer’s instruction, and RNA content was quantified by NanoDrop 2000 Spectrophotometer (Thermo Fisher Scientific, Wilmington, DE). Only 1 μg of total RNA, after DNase I treatment (Boehringer Manheim), was used per sample for the synthesis of cDNA. First strand cDNA template was synthesized using Oligo-dT as primers and Multiscribe reverse transcriptase (Takara).

### Isolation of full-length MADS-box B class genes of *T*. *erecta*

Based on the transcriptome sequence (accession number SRP066084) [29], five partial cDNA clones with different sequences were selected as B class genes of *T*. *erecta*, named as *TePI*, *TeAP3*-*1*, *TeAP3*-*2*, *TeTM6*-*1*, and *TeTM6*-*2*, respectively. The protein coding sequence (CDS) was predicted by using NCBI blast 2.2.28+. In order to verify the accuracy of CDS, primers set for the clones with complete coding sequences were designed in the 3’ and 5’ terminal region using the Primer 5 software (S1 Table). The cDNA from flower buds 5 mm in diameter were used as material to isolate the CDS of B class genes. The reaction mixture contained 2.5 μl of the first strand cDNA as template, 100 μM of dNTPs, 0.2 μM primer, 50 mM KCl, 10 mM Tris-HCl (pH 8.3), 1.5 mM MgCl_2_, 1 unit of Taq DNA polymerase (TaKaRa Biotechnology, Dalian, China), and double-distilled H_2_O to a total volume of 50 μl. The PCR procedure was initiated with pre-denaturation at 94°C for 4 min, followed by 35 cycles of amplification at 94°C for 30 s, 58°C for 30 s and 72°C for 1 min, and a final extension at 72°C for 10 min. The PCR products were cut from the 1.2% agarose/EtBr gel, cloned in the pMD18-T Vector (TaKaRa Biotech, Dalian, China) and the fragments were sequenced using universal M13F primer (Shanghai Invitrogen Biological Technology Co., Ltd.). Sequenced data were analyzed using the DNAMAN software (Lynnon Corporation). The full-length sequences of *TePI*, *TeAP3*-*1*, *TeAP3*-*2*, *TeTM6*-*1*, and *TeTM6*-*2* have been deposited in GenBank (http://www.ncbi.nlm.nih.gov) under accession number KU696419, KU696420, KU696421, KU696422, and KU696423, respectively.

### Analysis of cDNA sequences and construction of phylogenetic tree

The cDNA sequences of five B class genes were used to search for homologous sequences via blast in the National Center for Biotechnology Information. A total of 37 corresponding nucleotide and amino acid sequences of class B MADS-box factors were downloaded from the NCBI GenBank. The cDNA sequences of the B class genes were analyzed using the DNAMAN software. The predicted amino acid sequences of the B class genes of *T*. *erecta* and other plant species were used for phylogenetic analysis. The multiple sequence alignment was performed using software Clustal X 1.83. A phylogenetic tree was constructed using the maximum likelihood analysis method provided by the MEGA 3.0 software with the model generated by default. The phylogenetic tree was tested with a bootstrap of 1000 replicates to ascertain the reliability of a given branch pattern.

### Expression of five MADS-box B class genes by quantitative Real-time PCR

To analyze the expression of five B class genes in *T*. *erecta*, first-strand cDNAs from the samples of roots, tender stems, fresh leaves, different sizes of flower buds (1 mm, 3 mm, 5 mm and 7 mm in diameter, respectively), sepals, petals and pistils of ray and disk florets, stamens of disk florets, receptacles, bracts, and ovaries of opened flowers were used as templates. Quantitative Real-time PCR was carried out using an SYBR Primix Ex Taq kit (TaKaRa, Dalian, China) according to manufacturer’s instructions and analyzed in the ABI 7500 Real-time System (Applied Biosystems, USA). The levels of gene expression were calculated by ABI Prism 7500 Sequence Detection System Software (Applied Biosystems, USA). Real-time PCR was performed with specific primers of target genes and primers of housekeeping gene (*beta-actin*) (S1 Table) and the products were amplified in 20 μl reaction volumes containing 2 μl template of the reaction mixture, 10 μl 2 × SYBR Green Master Mix, 2 μl forward primer, 2 μl reverse primer (10 μmol·μl^-1^ for primers) and double-distilled water. The PCR was carried out with the following cycling parameters: heating at 95°C for 2 min, 40 cycles of denaturation at 95°C for 10 s, annealing at 56°C for 20 s, and extension at 72°C for 35 s. Real-time PCR was performed in four experimental replicates for each sample and the expression values obtained were normalized against *beta*-*actin*. Data are shown as mean values ± standard deviation. Analysis of the relative gene expression data was conducted using the 2^−ΔΔCt^ method.

### Yeast two-hybrid assays

The full length coding cDNA sequences of *TePI*, *TeAP3*-*1*, *TeAP3*-*2*, *TeTM6*-*1*, and *TeTM6*-*2* genes containing restriction sites at the 5’and 3’ ends were amplified by PCR from the sequenced clones using primers in S2 Table. The PCR products were introduced into the GAL4-based yeast two-hybrid vectors pGBKT7 (Clontech) and pGADT7-Rec (Clontech) and co-transformed into the AH109 yeast strain by the LiAc/DNA/PEG method according to the Yeast Protocols Handbook from Clontech (http://www.clontech.com). Co-transformed yeast cells were placed on selection plates with synthetic defined (SD) media lacking leucine (Leu) and tryptophan (Trp). Yeast double transformants were tested for interaction/activation on selective SD—Leu—Trp media. Positive interactions were confirmed by yeast growth in SD selective medium without Leu, Trp, histidine (His) and adenine (Ade). X-gal assay was performed for yeast cells grown on SD—Leu—Trp—His—Ade plates following instructions.

### Production of transgenic plants and phenotypic analysis of the transformants

The cDNA fragments containing the complete coding sequences of the five MADS-box B class genes of *T*. *erecta* were amplified using primers shown in S1 Table and cloned in a pMD18-T vector (Takara). Sequence accuracy and insection direction were confirmed by sequencing. After digestion with restriction enzymes (*Bam*HI and *Sal*I for *TePI*, *TM6*-*1*, and *TM6*-*2*; *Kpn*I and *Sal*I for *TeAP3*-*1*; *Xba*I and *Sal*I for *TeAP3*-*2*), the amplified fragments were subcloned into a binary vector pBI2300 which contained the CaMV35S promoter and the gene conferring kanamycin resistance. The vectors containing *35S*::*TePI*, *35S*::*TeAP3*-*1*, *35S*::*TeAP3*-*2*, *35S*::*TeTM6*-*1* and *35S*::*TeTM6*-*2* were introduced into the *Agrobacterium* strain EHA105 by the heat shock method, respectively. Leaf disk method for *Agrobacterium*-mediated transformation of *Nicotiana rotundifolia* was performed as described by Horsch et al. [30]. Transgenic *N*. *rotundifolia* plants at T_0_ generation were screened out from the non-transgenic line, and the presence of the transgene was confirmed by genomic PCR with 35S-F and specific primers (S1 Table). Transgenic and non-transgenic plants were grown in a specially assigned greenhouse for morphological comparisons following anthesis.

## Results

### Isolation and sequence analysis of five MADS-box B class genes of *T*. *erecta*

**Fig 1:**
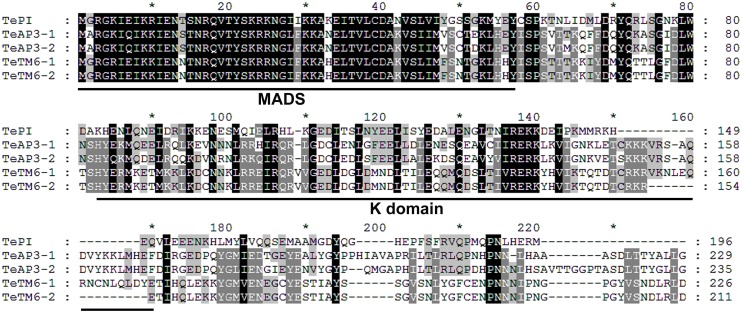
Comparison of the putative amino acid sequences of five MADS-box B class genes of *T*. *erecta*. The predicted amino acid sequences demonstrated that protein sequences of all the isolated B class genes of *T*. *erecta* have the typical MIKC-type domain structure. The MADS and K domains are indicated by black lines.

Based on the transcriptome sequence (accession number SRP066084) [29], full-length cDNA clones of five B class genes of *T*. *erecta* were isolated from a cDNA library constructed from flower buds 5 mm in diameter. These clones were named *TePI*, *TeAP3-1*, *TeAP3-2*, *TeTM6-1*, and *TeTM6-2*, respectively. The open reading frame (ORF) of cDNA sequences and predicted amino acid sequences were aligned in S1 Fig and Fig 1. The *TePI* transcript was predicted to have a 591 bp ORF and encode a protein made up of 196 amino acids. Two copies of *TeAP3*, *TeAP3*-*1* containing a 690 bp ORF and *TeAP3*-*2* containing a 708 bp ORF, encoded proteins made up of 229 and 235 amino acids, respectively. Two copies of *TeTM6*, *TeTM6*-*1* containing a 681 bp ORF and *TeTM6*-*2* containing a 636 bp ORF, encoded 226 and 211 amino acid proteins, respectively. The putative amino acid sequence of *TeAP3*-*2* gene was homologous to the *TeAP3*-*1* gene with 80% homology. Besides, the *TeTM6*-*2* was a partial fragment of *TeTM6*-*1* and most of their sequences were identical except additional 45 nucleotides of cDNA sequence and fifteen amino acids in the K domain of amino acid sequence in *TeTM6*-*1*.

**Fig 2:**
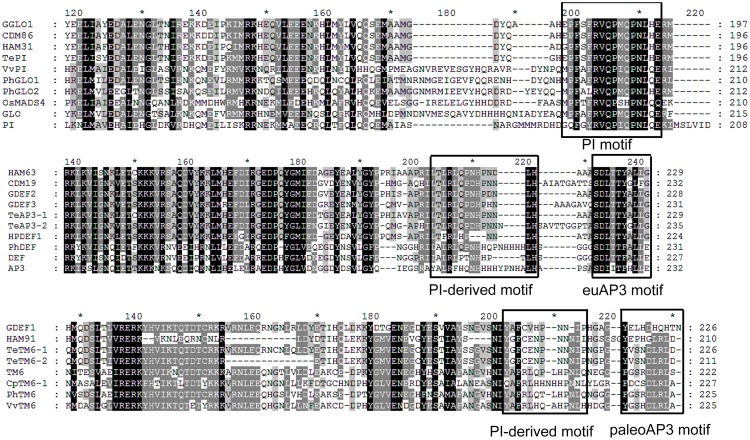
Comparison of the C-terminal region of amino acid sequences for MADS-box B class genes. TePI protein has the highly conserved sequence PI-motif in the C-terminal region. TeAP3-1 and TeAP3-2 have a PI-derived motif and a conserved euAP3 motif in the C-terminal region. TeTM6-1 and TeTM6-2 shared a PI-derived motif and a characteristic ancestral paleoAP3 motif instead of a euAP3 motif at the C-terminal end. In addition to the five B class genes of *T*. *erecta*, GenBank accession numbers of other PI/AP3-homologous genes were included in Fig 3.

The predicted amino acid sequences demonstrated that the protein sequences of the five B class genes of *T*. *erecta* had the typical MIKC-type domain structure, namely, the MADS-domain, I-region, K-domain and C-region (Fig 1). Furthermore, TePI protein had the highly conserved PI-motif between amino acid residues 197 and 211 within the C-terminal region (Fig 2a). Moreover, TeAP3-1 and TeAP3-2 proteins had a PI-motif-derived region and a conserved euAP3 motif in the C-terminal region (Fig 2b). Similarly, TeTM6-1 and TeTM6-2 proteins shared a PI-motif-derived region and a characteristic ancestral paleoAP3 motif instead of the euAP3 motif at the C-terminal (Fig 2c).

### Phylogenetic analyses

**Fig 3:**
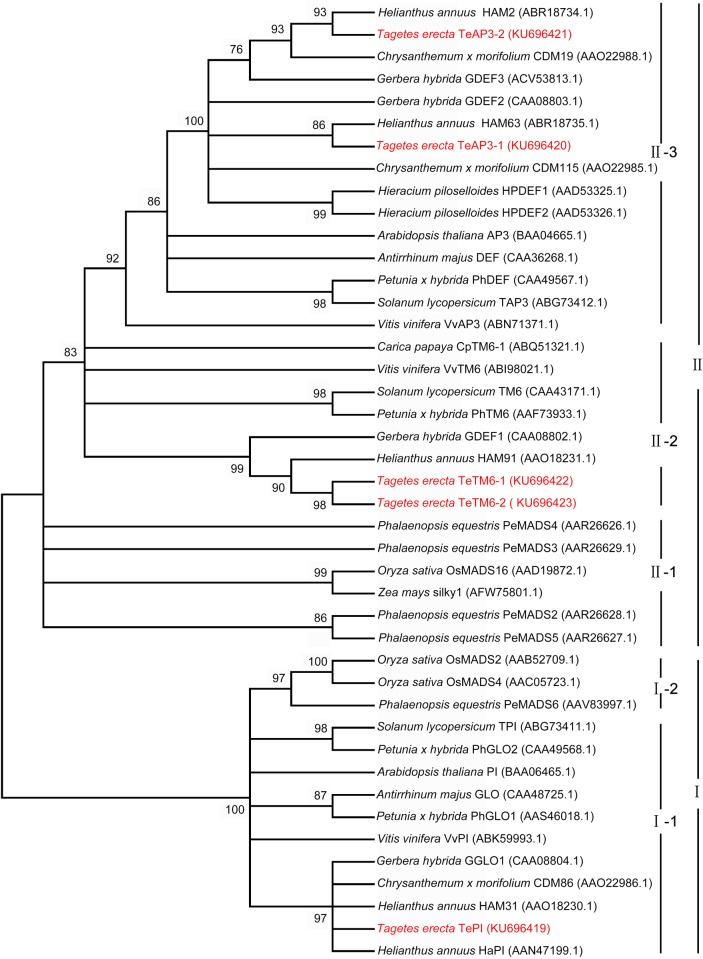
Phylogenetic tree calculated from the amino acid sequence of forty-two MADS-box B class genes. A phylogenetic tree was constructed based on the amino acid sequences using the maximum likelihood analysis method. In addition to *T*. *erecta*, the full-length amino acid sequences of MIKC domains were selected from 37 known *PI/AP3*-homologous genes. The phylogenetic tree showed that the TePI protein was classified into the PI/GLO subfamily, TeAP3-1 and TeAP3-2 placed within the euAP3clade, while TeTM6-1 and TeTM6-2 placed within the TM6 clade. Besides, TeAP3-1 and TeAP3-2 were divided into two paralogous groups in the Asteraceae-specific subgroup of euAP3 clade. Ι: PI/GLO subfamily; Ι-1: PI/GLO in eudicot species; Ι-2: PI/GLO in monocot species; ΙΙ: AP3/DEF subfamily; ΙΙ-1: AP3/DEF in monocot species; ΙΙ-2: TM6 clade in eudicot species; ΙΙ-3: euAP3 clade in eudicot species.

To determine the phylogenetic relationship of the MADS-box B class genes of *T*. *erecta* with other species, a phylogenetic tree was constructed using full-length amino acid sequences of MIKC domains from 37 known *PI/AP3*-homologous genes and the five MADS-box B class genes of *T*. *erecta*. All sequences used with their GenBank accession numbers and respective species were listed in Fig 3. The maximum likelihood analysis demonstrated that the B class gene family could be divided into *PI*/*GLO* and *AP3*/*DEF* subfamilies (Fig 3). *T*. *erecta* belongs to the Asteraceae family and all members in this family were gathered in the core eudicots group, separating themselves from the monocot species. The phylogenetic tree revealed that the *TePI* gene was classified into the *PI*-like genes, while *TeAP3*-*1*, *TeAP3*-*2*, *TeTM6*-*1*, and *TeTM6*-*2* were all grouped into the clade of *AP3*-like genes. More specifically, *TeAP3*-*1* and *TeAP3*-*2* were classified into the eu*AP3* clade, while *TeTM6*-*1* and *TeTM6*-*2* were classified into the *TM6* clade. Furthermore, we found that *TeAP3*-*1* and *TeAP3*-*2* were divided into two paralogous groups in the Asteraceae-specific subgroup of the eu*AP3* clade.

### Expression analysis of five MADS-box B class genes in *T*. *erecta*

**Fig 4:**
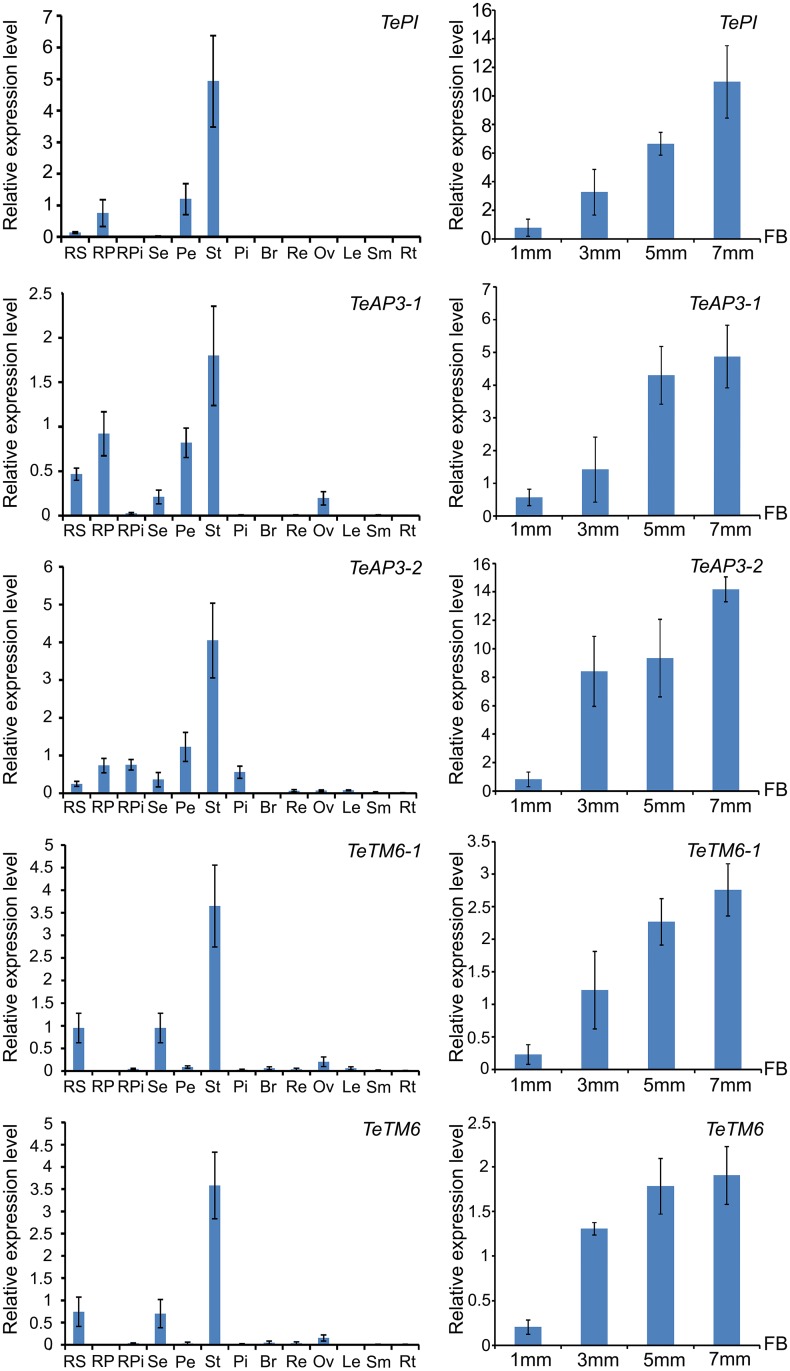
Expression levels of B class genes in different tissues, organs and different development stages of flower buds of *T*. *erecta*. The relative expression level showed that strong expression of *TePI* was detected in stamens of disk florets, and weak expression in petals of disk and ray florets along with little expression in sepals of ray florets and no expression was detected in pistils of ray florets, sepals of disk floret, bracts, receptacles and vegetative organs. *TeAP3-1* was expressed in sepals and petals of ray and disk florets, stamens of disk florets, and ovaries, but only weakly expressed in pistils of ray florets. *TeAP3-2* was ubiquitously expressed in sepals, petals, stamens and pistils of the florets. Strong expression of *TeTM6-1* was detected in stamens of disk florets, weak expression was detected in sepals of disk and ray florets, ovaries, and petals of disk florets. The expression levels of *TeTM6-2* and *TeTM6-1* were detected in one Real-time PCR reaction (*TeTM6*), no expression of *TeTM6-1* and *TeTM6-2* could be detected in petals of ray florets. The relative expression levels of B class genes in different sizes of flower buds revealed that the expression level increased with the size of flower buds in *T*. *erecta*. RS: sepal of ray floret; RP: petal of ray floret; RPi: pistil of ray floret; Se: sepal of disk floret; Pe: petal of disk floret; St: stamen of disk floret; Pi: pistil of disk floret; Br: bract; Re: receptacle; Ov: ovary; Le: leaf; Sm: stem; Rt: root; FB: size of flower bud; 1 mm: flower buds 1 mm in diameter; 3 mm: flower buds 3 mm in diameter; 5 mm: flower buds 5 mm in diameter; 7 mm: flower buds 7 mm in diameter.

The expression patterns of the five B class genes in different floral organs and tissues of *T*. *erecta* were analyzed by quantitative Real-time PCR. The expression levels of these genes increased significantly as flower buds grew bigger (Fig 4). Relative expression levels showed that these MADS-box B class genes were expressed at significant levels exclusively in stamens of the disk florets, but little or no expression was detected in bracts, receptacles or vegetative organs (Fig 4). Taking the example of *TePI*, strong expression was detected in stamens of disk florets, weak expression was detected in petals of disk and ray florets, little expression was detected in sepals of ray florets and no expression was detected in pistils of ray florets, sepals of disk florets, bracts, receptacles and vegetative organs. This result indicated that *TePI* was predominantly involved in the formation of stamen and petal in *T*. *erecta*. *TeAP3*-*1* was strongly expressed in sepals and petals of disk and ray florets, stamens of disk florets and ovaries, but weakly expressed in the pistils of ray florets. Besides, the transcript signals were hardly detected in pistils of disk florets, bracts, receptacles and vegetative organs. Expression pattern of *TeAP3*-*2* was similar to *TeAP3*-*1*, but its expression level in pistils of ray and disk florets was higher than that of *TeAP3*-*1*, no expression was detected in ovaries. Strong expression of *TeTM6*-*1* was detected in stamens of disk florets, weak expression was detected in sepals of disk and ray florets, ovaries, petals of disk florets, whereas no expression could be detected in petals of ray florets. As most of the sequences of *TeTM6*-*2* were identical to that of *TeTM6*-*1*, it was difficult to distinguish the expression level of *TeTM6*-*2* from *TeTM6*-*1*. Instead, we detected the expression levels of *TeTM6*-*1* and *TeTM6*-*2* in one Real-time PCR reaction and found that no expression of *TeTM6*-*2* could be detected in petals of ray florets.

### Protein interactions of five MADS-box B class proteins

**Fig 5:**
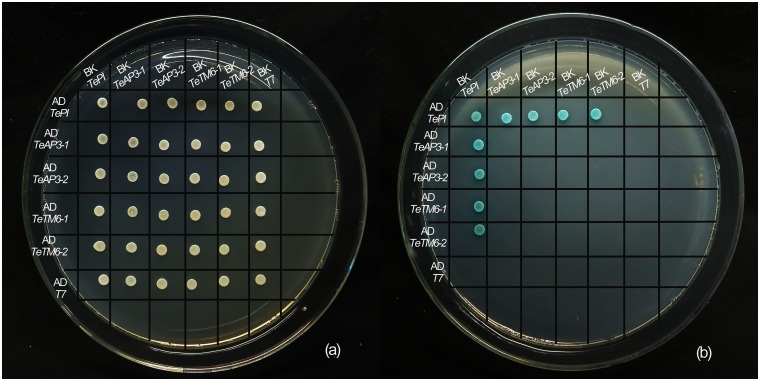
Yeast two-hybrid interactions of five MADS-box B class proteins of *T*. *erecta*. The PCR products were introduced into the GAL4-based yeast two-hybrid vectors pGADT7 vector (AD) and pGBKT7 vector (BK) and co-transformed into the AH109 yeast strain. (a) Co-transformed yeast cells were plated on selection plates SD / -Trp-Leu-solid medium. (b) Yeast growth on selection plates SD / -Trp-Leu-His-Ade / X-α-GAL solid medium showed that TePI protein could interact with other four AP3/TM6-like proteins. No homodimer or interaction between the AP3/TM6-like proteins was observed in *T*. *erecta*. Besides, we observed homodimerization in TePI proteins.

To determine the protein interactions of the B class proteins *in vitro*, yeast two-hybrid assays were performed between the B class proteins. The results of two-hybrid analysis revealed that the TePI protein could interact with all of the four AP3-type proteins (Fig 5) and homodimerization could be observed in TePI proteins. No homodimer or other interaction between the AP3-type members was observed in *T*. *erecta*.

### Constitutive expression of five MADS-box B class genes of *T*. *erecta* in *N*. *rotundifolia*

**Fig 6:**
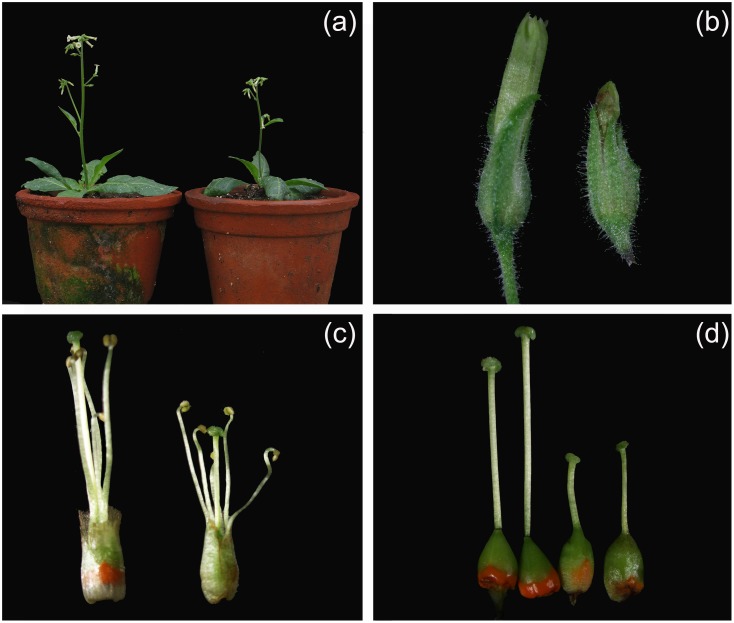
Phenotypic comparison between non-transgenic and transgenic *N*. *rotundifolia* lines with overexpression of *35S*::*TePI*. (a) The non-transgenic (left) and transgenic plant of *35S*::*TePI* (right). (b) Flower buds from the non-transgenic (left) and a transgenic plant of *35S*::*TePI* (right). (c) Stamens and pistils from the non-transgenic (left) and a transgenic plant of *35S*::*TePI* (right). (d) Carpels from the non-transgenic (left two) and a transgenic plant of *35S*::*TePI* (right two). The ovary surface of the transformants was rough. The petal, stamen and pistil of transgenic plants were significant shorter than the non-transgenic plants.

To determine the potential role of B class genes of *T*. *erecta* in the floral development, the identified five class B genes were ectopically expressed in *N*. *rotundifolia* through *Agrobacterium*-mediated transformation. At least 21, 17, 19, 18, and 17 independently transformed *N*. *rotundifolia* lines of *35S*::*TePI*, *35S*::*TeAP3*-*1*, *35S*::*TeAP3*-*2*, *35S*::*TeTM6*-*1* and *35S*::*TeTM6*-*2* were obtained by PCR detection, respectively. The non-transgenic plants and transformants of the T_0_ generation were selected for morphological comparisons. Results showed that none of the transgenic *N*. *rotundifolia* lines of *35S*::*TeAP3*-*1*, *35S*::*TeAP3*-*2*, *35S*::*TeTM6*-*1* or *35S*::*TeTM6*-*2* showed any obvious morphological changes from the non-transgenic line. For transgenic lines of *35S*::*TePI*, five out of 21 *N*. *rotundifolia* plants showed altered floral morphology as compared with the non-transgenic line (Fig 6). There was a decrease in plant height of the transformants compared with the non-transgenic plants (Fig 6a, Table 1), but no significant differences were observed in terms of crown size, leaf length and leaf width (Table 1). The most obvious floral changes were detected in the second, third and forth whorls of the flower. The petal (Fig 6b), stamen (Fig 6c) and style (Fig 6d) of transformants were significantly shorter than the non-transgenic line, whereas the ovaries of transformants became larger (Table 1). In addition, in the non-transgenic plants, the ovary was conical shaped and the surface was smooth, but in transgenic plants, the ovary was football-like shaped and the surface was rough (Fig 6d).

**Table 1:** Phenotypic analysis of non-transgenic and transgenic *N*. *rotundifolia* lines of *35S*::*TePI* unit:cm.

Traits	Non-transgenic lines	Transgenic lines of *35S*::*TePI*
Number of plant	15	5
Plant height	20.61±1.43*	15.83±1.13
Crown size	18.02±1.27	17.20±1.56
Leaf length	9.30±0.71	8.74±0.57
Leaf width	3.70±0.34	3.53±0.23
Flower height	1.27±0.14*	1.03±0.11
Flower diameter	0.66±0.03	0.62±0.03
Stamen length	1.08±0.04*	0.80±0.07
Style length	0.87±0.06*	0.40±0.05
Ovary height	0.25±0.03	0.32±0.02*

*Indicated significant difference by Student *t* test (P<0.001)

## Discussion

The B class genes were divided into *AP3/DEF* and *PI/GLO* subfamilies [21, 22, 31, 32]. Based on the completely divergent C-terminal motifs, the eu*AP3* and *TM6* lineages were recognized as the clades in the *AP3/DEF* subfamily in a number of higher eudicot species [18]. In this study, five MADS-box B class genes were isolated and characterized in *T*. *erecta*. Sequence comparisons and phylogenetic analyses indicated that there were two eu*AP3*-like genes in *T*. *erecta*, which was consistent with findings in many other Asteraceae species [28, 33]. Broholm et al. [27] and Barker et al. [34] concluded that the paralogous pair of eu*AP3* lineage genes was a common origin, possibly from the genome-wide duplication at the base of the Asteraceae lineage. Furthermore, there were two *TM6*-like genes in *T*. *erecta*, and the *TeTM6*-*2* was a partial fragment of *TeTM6*-*1* and there seemed to be no difference between their protein interaction capacities, indicating that *TeTM6*-*1* or *TeTM6*-*2* was completely functionally redundant. Meanwhile, our study proved that there was only one *TePI* gene belonging to the *PI/GLO* subfamily in *T*. *erecta*. This result agreed with the finding in *G*. *hybrida* [27] but contrasted with findings in *H*. *annuus* [28] and *Chrysanthemum* x *morifolium* [35] in which two and three *PI/GLO*-like genes were found, respectively.

According to the classical ABC model, B class genes are essential for the development of the second and third whorls of flowers [21, 22, 32, 36]. Thus, it was expected that the B class genes would predominantly expressed in petals and stamens [37]. However, expression studies of MADS-box B class genes in several eudicot species revealed that B-function regulation varied within the eudicot lineages [18, 37]. In this study, apart from petals and stamens, *TeAP3*-*1* was also expressed in sepals, ovaries, and pistils of ray florets, and *TeAP3*-*2* was also expressed in sepals and pistils of ray and disk florets. There are many studies focusing on the broader expression domains of eu*AP3* genes amongst different plant species. The eu*AP3* genes were not only expressed in petal and stamen, but also in sepal, carpel and all the other floral organs in *A*. *majus* [31, 38], *Nicotiana glauca* [39], *Petunia hybrida* [40], and *Solanum lycopersicum* cv. Micro-Tom [41]. Besides, the eu*AP3* genes were also expressed in the ovules in *A*. *thaliana* [22] and *Medicago truncatula* [42]. In *G*. *hybrida*, *GDEF2* and *GDEF3* transcripts were detected throughout all of the four whorls of floral organs, as well as in vegetative organs such as leaves and petioles. *GDEF2* was also expressed in scapes, ovules, leaves and roots [27, 43]. In *Hieracium piloselloides*, the expression of *HpDEF* was detected along with ovule development before anthesis [44]. *TeTM6*-*1* was strongly expressed in the stamens of disk florets, weakly expressed in sepals of disk and ray florets, ovaries and petals of disk florets. No expression of *TeTM6*-*1* and *TeTM6*-*2* were detected in the petals of ray florets. Similar results were reported in the expression of *GDEF1* in *G*. *hybrida* where *GDEF1* transcripts were detected in all of the four floral whorls in disk flowers but not in the petals of ray flowers at comparable developmental stages [27, 43]. Similar phenomenon was also reported in *P*. *hybrida*, where *PhTM6* was mainly expressed in the developing stamens but played no role in petal development [45]. Vandenbussche et al. [37] concluded that *PhTM6* behaved more like a C-function gene than a B-function gene. Besides, the *TeAP3*-*1*, *TeTM6*-*1* and *TeTM6*-*2* genes were expressed in the ovary, indicating that these genes might have a role in ovule development.

To further investigate the function of *TePI*, *TeAP3*-*1*, *TeAP3*-*2*, *TeTM6*-*1* and *TeTM6*-*2*, the five B class genes connected with the 35S promoter were transformed into *N*. *rotundifolia* through *Agrobacterium*-mediated transformation. None of the transgenic plants of *35S*::*TeAP3*-*1*, *35S*::*TeAP3*-*2*, *35S*::*TeTM6*-*1* or *35S*::*TeTM6*-*2* showed obvious morphological changes from the non-transgenic line. Similar phenomena occurred in many other species, such as *Rosa rugosa* [46], *Narcissus tazetta* var. *Chinensis* [47], *Chimonanthus praecox* [48] and *Prunus mume* [49]. It seemed that *TeAP3*-*1*, *TeAP3*-*2*, *TeTM6*-*1* and *TeTM6*-*2* could not regulate floral organ development independently, when ectopically expressed in *N*. *rotundifolia*. By contrast, the transgenic plants of *35S*::*TePI* showed obvious changes in floral morphology as the petal, stamen and pistil were significantly shorter than the non-transgenic plants and the surface of the abnormally shaped ovary was rough, indicating that the over-expression of *TePI* could affect floral organ development in *N*. *rotundifolia*. The ectopic expression of *PI*-like genes may have different effects in the floral organ development of transformants. Over-expression of *Platanus acerifolia PaPI2a* gene in transformed *Nicotiana tabacum* led to elongated sepals, shorter pistils and abnormal ovaries covered with trichomes [50]. Over-expression of *PeMADS6* of *Phalaenopsis equestris* in *A*. *thaliana* resulted in petaloid sepals [51].

According to the previous studies, AP3/DEF and PI/GLO proteins are functional partners and interact with each other to form obligate heterodimers for DNA binding *in vitro* and to regulate gene expression by binding to the CArG motif of their promoters [8, 52]. In the present study, TePI protein could form heterodimers with all the other four B class proteins TeAP3-1, TeAP3-2, TeTM6-1, and TeTM6-2. No homodimer or other interaction was observed between the euAP3 or TM6 clade members. But interestingly, TePI protein could form a homodimer. B class proteins have been shown to form homodimers or heterodimers in monocot species and function as AP3/PI heterodimers in eudicot species. Some of the DEF/AP3-like MADS-box proteins from monocots have the ability to form homodimers, such as LMADS1 of *Lilium longiflorum* [53], PeMADS4 and PeMADS6 of *P*. *equestris* [54], OMADS5, OMADS3 and OMADS9 of *Oryza sativa* [55]. For eudicot species, Roque et al. [42] observed homo-dimerization of MtPI protein in *M*. *truncatula*, but the underlying mechanism is not clear and requires further studies.

Genetic male sterile lines have been utilized in the F_1_ hybrid production of *T*. *eracta* for many years [56, 57]. So far, however, the molecular mechanism of the male sterility trait in *T*. *eracta* remains unclear. Many reports indicated that the male sterility trait in *T*. *eracta* was controlled by a recessive gene [58, 59], but no related functional gene has been characterized yet. He et al. [59] found that the petals of the ray and disk florets of the male sterile plant of *T*. *erecta* developed to sepal-like, while the stamens developed to carpel-like structures, they concluded that the spontaneously derived genetic male sterility trait was the result of homeotic conversion of floral organs and assumed that the homeotic conversion in *T*. *erecta* might be due to the mutation of B class MADS-box genes. In this study, only the *TePI* gene was predominantly expressed in the petals of ray and disk florets and stamens of disk florets, while the other four *AP3* and *TM6* genes were not only expressed in petals and stamens, but also in other floral organs. And among all transgenic *N*. *rotundifolia* plants of five B class genes of *T*. *erecta*, only transgenic plants of *35S*::*TePI* showed altered floral morphology. Thus, it is assumed that the male sterility traits of *T*. *erecta* might be associated with *TePI*, which is worthy of further investigation.

## Supporting Information

S1 Fig Sequence alignment of cDNA of five MADS-box B class genes of *T*. *erecta*. (TIF)

S1 Table List of primers in this study. (DOCX)

S2 Table Specific primers of five MADS-box B class genes of *T*. *erecta* containing restriction sites for the yeast two-hybrid assay. (DOCX)
