# Supplementary figures and images for: Characterization and Functional Analysis of Five MADS-Box B Class Genes Related to Floral Organ Identification in Tagetes erecta
Source: PLoS One. 2017 Jan 12;12(1):e0169777. doi: 10.1371/journal.pone.0169777 (PMC5231280; doi:10.1371/journal.pone.0169777)

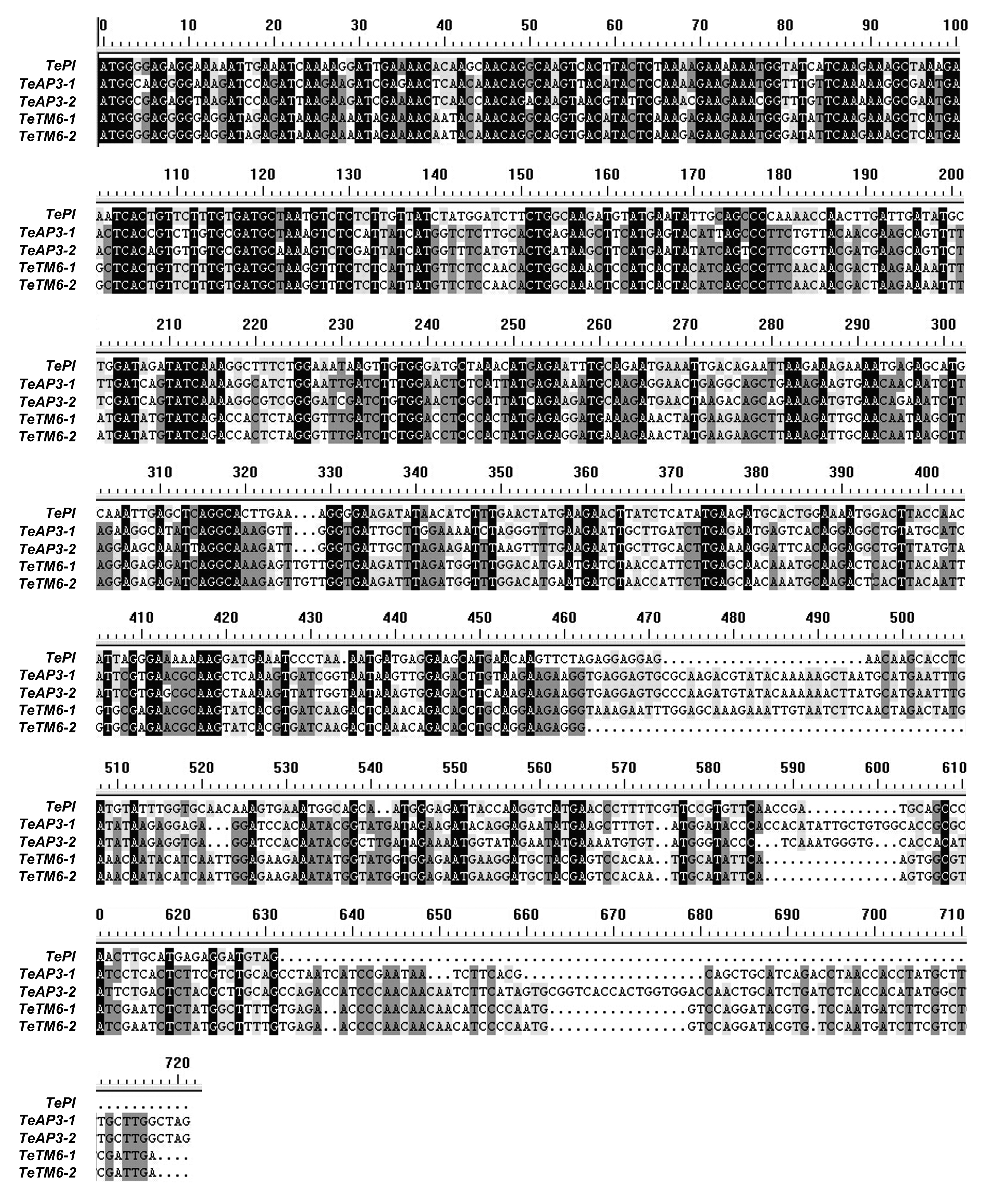

Supplement: S1 Fig — (TIF) [file pone.0169777.s001.tif]
