# Supplementary material for: Characterization and Functional Analysis of Five MADS-Box B Class Genes Related to Floral Organ Identification in Tagetes erecta
Source: PLoS One. 2017 Jan 12;12(1):e0169777. doi: 10.1371/journal.pone.0169777 (PMC5231280; doi:10.1371/journal.pone.0169777)
Supplement: S1 Table — (DOCX) [file pone.0169777.s002.docx]

**S1 Table List of primers in this study**

| Primers | Sequence (5' to 3') | Remarks |
| --- | --- | --- |
| *TePI*-cDNA-F | GCCAGTTGTGTTGGACTTGTCA | Forward (F) and reverse (R) primers for the clones with complete coding sequences of target genes |
| *TePI*-cDNA-R | GGACGAGTGACCCTAGCAAATC |  |
| *TeAP3-1*-cDNA-F | CCCTTTACACACATTTGATCCC |  |
| *TeAP3-1*-cDNA-R | CACGCATAAACAATCAGTCACAA |  |
| *TeAP3-2*-cDNA-F | ACAAAATCAATGGCGAGAGGTA |  |
| *TeAP3-2*-cDNA-R | ATCATGAGTCAACCAAACGAGG |  |
| *TeTM6*-cDNA-F^1^ | AAAGACAAGTAAGATCGAGATGGGG |  |
| *TeTM6*-cDNA-R | TGATTTAAAAACTCAATCGAGACGA |  |
| *ACT*-RT-F | GGGAAATGAATGCCAAAGCCAAG | Forward (F) and reverse (R) primers of *beta-actin* (*ACT*) genes and target genes in Real-time PCR (RT) |
| *ACT*-RT-R | AAGACTTCACAACCACTCTCCAACT |  |
| *TePI*- RT-F | GAAATCCCTAAAATGATGAGGAAGC |  |
| *TePI*- RT-R | ATCGGTTGAACACGGAACGAAAAG |  |
| *TeAP3-1*- RT-F | CAAGCTCAAAGTGATCGGTAATAAG |  |
| *TeAP3-1*- RT-R | ATATGTGGTGGGTATCCATACAAAG |  |
| *TeAP3-2*- RT-F | TACTGTTGGCTTATTCATAACCTCG |  |
| *TeAP3-2*- RT-R | CAGTATCTGAAAACACAAAACAAAACC |  |
| *TM6-1*- RT-F | AGGAAGAGGGTAAAGAATTTGGAGC |  |
| *TM6-1*- RT-R | TGGACTCGTAGCATCCTTCATTCTC |  |
| *TM6*-RT-F^2^ | ATGGTGGAGAATGAAGGATGCTAC |  |
| *TM6*-RT-R | CATGATTTAAAAACTCAATCGAGACG |  |
| 35S-F | ACGCACAATCCCACTATCCTTC | *CaMV35S* promoter |

^1^*TeTM6*-cDNA-F and *TeTM6*-cDNA-R were used to amplify the complete coding sequences of *TeTM6-1* and *TeTM6-2* genes

^2^*TM6*-RT-F and *TM6*-RT-R were applied to detect the expression levels of *TeTM6-1* and *TeTM6-2* genes simultaneously.
