# Supplementary material for: Characterization and Functional Analysis of Five MADS-Box B Class Genes Related to Floral Organ Identification in Tagetes erecta
Source: PLoS One. 2017 Jan 12;12(1):e0169777. doi: 10.1371/journal.pone.0169777 (PMC5231280; doi:10.1371/journal.pone.0169777)
Supplement: S2 Table — (DOCX) [file pone.0169777.s003.docx]

**S2 Table Specific primers of five MADS-box B class genes of *T. erecta* containing restriction sites for the yeast two-hybrid assay**

| Primer^1^ | Sequence (5’ to 3’) | Restriction sites |
| --- | --- | --- |
| *TePI*-ABD-F | AGTGAATTCATGGGGAGAGGAAAAATTGAA | *Eco*RI |
| *TePI*-ABD- R | AGTGGATCCCTACATCCTCTCATGCAAGTT | *Bam*HI |
| *TeAP3-1*-ABD- F | AGTGAATTCATGGCAAGGGGAAAGATCCA | *Eco*RI |
| *TeAP3-1*-AD- R | AGTGAGCTCCTAGCCAAGCAAAGCATAGGT | *Sac*I |
| *TeAP3-1*- BD-R | AGTGTCGACCTAGCCAAGCAAAGCATAGGT | *Sal*I |
| *TeAP3-2*- ABD-F | AGTGAATTCATGGCGAGAGGTAAGATCCA | *Eco*RI |
| *TeAP3-2*- AD-R | AGTGAGCTCCTAGCCAAGCAAGCCATATGT | *Sac*I |
| *TeAP3-2*- BD-R | AGTCTGCAGCTAGCCAAGCAAGCCATATGT | *Pst*I |
| *TeTM6*- ABD-F^2^ | AGTGAATTCATGGGGAGGGGGAGGATAGA | *Eco*RI |
| *TeTM6*- ABD-R | AGTGGATCCTCAATCGAGACGAAGATCAT | *Bam*HI |

^1^The naming rule for primers is gene name - relative vector – forward (F) / reverse (R). AD: The restriction sites of the primers were included in the pGADT7-Rec vector; BD: The restriction sites of the primers were included in the pGBKT7 vector; ABD: The restriction sites of the primers were included both in the pGADT7-Rec and pGBKT7 vectors.

^2^*TeTM6*-ABD-F and *TeTM6*-ABD-R were used to amplify the full-length coding sequences of *TeTM6-1* and *TeTM6-2* genes.
